# Family Presence Restrictions and Telemedicine Use in Neonatal Intensive Care Units during the Coronavirus Disease Pandemic

**DOI:** 10.3390/children8070590

**Published:** 2021-07-12

**Authors:** Mio Ozawa, Haruyo Sakaki, Xianwei Meng

**Affiliations:** 1 Division of Nursing Science, Graduate School of Biomedical & Health Sciences, Hiroshima University, 1-2-3 Kasumi, Minami-ku, Hiroshima 734-8553, Japan; 2 Graduate School of Nursing, International University of Health and Welfare, 4-1-26 Akasaka, Minato-ku, Tokyo 107-8402, Japan; haruyo@iuhw.ac.jp; 3 Center for Baby Science, Doshisha University, 4-1-1 Kizugawadai, Kizugawa, Kyoto 619-0225, Japan; mokeni1211@gmail.com or; 4 Comparative and Developmental Psychology, Graduate School of Human Sciences, Osaka University, 1-2 Yamadaoka, Suita, Osaka 565-0871, Japan

**Keywords:** NICU design, hospital restriction policy, neonatal intensive care unit, parent, telemedicine

## Abstract

We aimed to describe parental presence policy and telemedicine use in Japanese neonatal intensive care units (NICUs) before and during the coronavirus disease (COVID-19) pandemic. This cross-sectional study was performed through an online survey in 110 level III units from 19 November 2020 to 18 December 2020. Nurses’ evaluation of the current situation (during COVID-19) was compared with their retrospective pre-COVID-19 (December 2019) evaluation. Responses were received from 52 NICUs distributed across all regions in Japan. The median allowed parental presence time decreased from 12 h to 1 h, and 29 NICUs allowed entry of parents simultaneously during COVID-19. There was an increase in the number of units providing telemedicine through telephone and online visits during COVID-19 compared to that before COVID-19 (from 2% to 19%). The hybrid design NICUs, with 11–89% of beds in single-patient rooms, allowed a longer parental presence time in the NICUs than those with ≥90% of beds in multi-bed rooms. The number of units implementing kangaroo care decreased during COVID-19 compared to that before COVID-19. The need for telemedicine increased among Japanese NICUs to mitigate the adverse effect of parental restriction and limited physical contact due to the COVID-19 pandemic.

## 1. Introduction

The coronavirus disease (COVID-19) pandemic continues to ravage the world. Since the first reported case of COVID-19 was confirmed in Japan on 16 January 2020, 794,884 cases and 14,738 deaths have been reported as of 29 June 2021 [1]. In Japan, most inpatient medical facilities imposed restrictions on family members’ visits to admitted patients, as the government declared a temporary nationwide closure of all elementary and junior high schools on 2 March 2020, followed by a state of emergency declaration on April 7. Family members were not allowed to gain access into the ward except at the time of patients’ admission and discharge and delivery of the necessities required in the hospital.

Restrictions of patient’s parents on admission may reduce face-to-face communication with NICU providers and the joint care for their children, leading to delays in preparing for discharge and prolonging hospital stays. It was reported that parents with restricted access to the NICU due to the novel COVID-19 outbreak experienced negative feelings, such as sadness and anger [2]. Thus, measures of parental restrictions should be carefully considered by providing alternatives that will prevent the adverse effects of these restrictions. Previous studies have suggested that NICU design (in which the single-family room had the loosest entrance restrictions for parents, followed by the hybrid design and the Open bay design with the strictest parental entrance restriction) [3], telemedicine introduction [4], and NICU visitation policy [5] may be useful means of mitigating the detachment of or adverse effects on parents. 

One study reported the introduction of parental video calls and NICU staff during COVID-19. They communicated by mobile phones, tablets, and personal computer (PC), while being in separate locations; parent satisfaction was reported with this method [4]. Although this study was conducted before COVID-19, no differences in the length of hospital stay were reported for children subjected to telemedicine between the Internet and control groups [6]. In Japanese NICUs, telephone is the most common device in telemedicine, and online devices (video/webcam, live videoconference, etc.) were not common before COVID-19. However, after the COVID-19 pandemic began, the development of an online visit system was designed by Osaka University Hospital; a donation of 30 million yen was collected for the plan until November 2020 [7]. Moreover, because medical devices may malfunction due to the electromagnetic waves of mobile phones, the use of smartphones is often prohibited in Japanese NICUs. There has been no report on the actual use of telemedicine in Japanese NICUs, and it is unknown what kind of device or system is used.

In this study, we described the effect of COVID-19 on parental presence and telemedicine to communicate between infants and parents or healthcare professionals and parents in NICUs, which are currently poorly defined. Additionally, we investigated the effects of parental restriction on infants’ care by parents and the association between delay discharge and NICU design, telemedicine, and parent visiting policy, which were reported to be a useful method for mitigating the negative impact on parents.

## 2. Materials and Methods

This study is a cross-sectional online survey of 110 level III NICUs [8] across Japan from 19 November 2020 to 18 December 2020. Participants retrospectively evaluated the past (in December 2019, i.e., before COVID-19) and current (during COVID-19) use of telemedicine. The survey involved the administration of questionnaires comprising 22 items (20 mandatory selective answer items and two optional descriptive answer items) (Appendix A). The questionnaire was drafted by MO and amended by MO, HS, and MX with reference to previous research [3] and the website [9]. The questionnaire was assessed for easy comprehension and appropriateness for the target audience by one neonatologist, two NICU nurses, one pediatrician, and one neonatal nurse researcher, and corrections were made where necessary. The average response time was 15 min. On 19 November 2020, the survey request letter was mailed to the head nurse of the NICU, requesting the nomination of the NICU nurse to participate in the online survey through PC or smartphones. A reminder was mailed on 4 December 2020. However, one of Shikoku region facilities informed us in writing that they would not participate in the study. Informed consent was obtained from the respondents, and the study was approved by the Hiroshima University Research Ethics Review Board (No.: E-2260).

The following three main approaches were used to analyze the data: sample descriptive statistics; comparisons of parental presence and telemedicine from before the spread of COVID-19 to after the start of the spread; and the association in delay discharge and NICU design, telemedicine, and parental visiting policy during COVID-19. For descriptive statistics, we used counts and percentages, as presented in tables and figures. For comparison before and during COVID-19, considering that the survey responses were quantitative, we used the Wilcoxon test for all pre–post comparisons and McNemar’s test or Cochran-Armitage test for all pre–post comparison in categorical variables. The association in categorical variables during COVID-19 was examined using Fisher’s exact tests. In this study, the data obtained from optional answer items were excluded from the analysis. The statistical software JMP Pro 15 (SAS Institute Japan, Tokyo, Japan) was used for all analyses; all significance levels were two-sided and set as 5%.

## 3. Results

Responses were received from 52 NICUs distributed across all regions, with a response rate of 48%. The response rates by region were 75%, 71%, 55%, 40%, 45%, 50%, 25%, and 35% in Hokkaido, Tohoku, Kanto, the Chubu, Kansai, Chugoku, Shikoku, and Kyushu, respectively. A greater percentage of the responses was obtained from the Kanto region, including the Tokyo metropolitan area, accounting for 31% of all responses. The 26 NICUs in the 13 prefectures with commuting restriction by the Government of Japan on 16 April 2020, due to many people with COVID-19, accounted for approximately half of the responses. The background information of the NICUs that responded in this study is shown in Table 1. Most NICUs were in the Open bay (≥90% of beds in multi-bedrooms) (*n* = 45), and few were in the Hybrid (11–89% of open beds in single-patient rooms) (*n* = 7). There were no NICUs in single-family rooms (≥90% of beds in single-patient rooms).

### 3.1. Parents’ and Family Visiting Screening and Restriction

**Figure 1:**
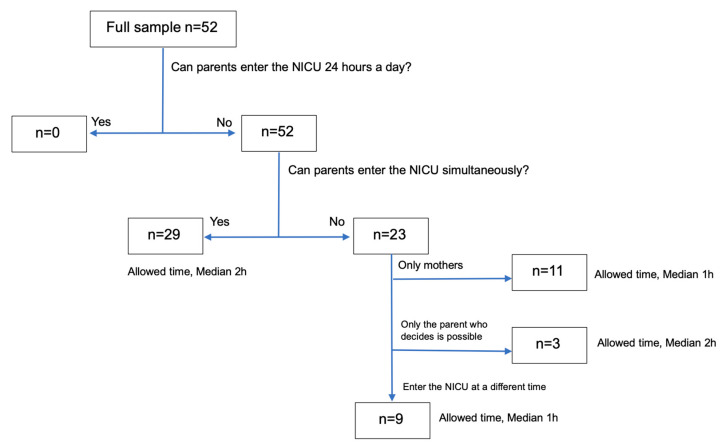
Overview of parental presence policies in the neonatal intensive care unit (NICU) during coronavirus disease (COVID-19).

During COVID-19, the NICUs began the restriction of parents’ entry; the median time of restriction commencement was 7 April 2020; by the end of April, more than 80% of NICUs had begun restrictions, and by late August, most NICUs restricted parents’ entry. The Kanto region NICU had the earliest restrictions implementation on 1 February 2020, while NICUs in the Hokkaido region started restrictions on 6 November 2020. The reasons for the restriction of parent’s entry are shown in Table 2. Table 3 shows NICU visiting screening before the COVID-19 outbreak (1 December 2019) and the response date. Migration history, poor physical condition, and temperature check-ups during COVID-19 increased compared to those before COVID-19. In all NICUs, there was a time restriction on parental visits. Parental visiting median time in the NICUs was limited to 1 h. The NICU where a nurse was confirmed to be positive for COVID-19 restricted parents from entering anytime. Furthermore, grandfathers, mothers, and siblings were prohibited from entering most NICUs. We found a variety of parental presence policies measured during COVID-19 (Figure 1).

All NICUs restricted parental presence, whereas 23 NICUs (44%) allowed only one parent at the bedside for the entire hospital stay, and a minority (21%) allowed only mothers into the NICU.

### 3.2. Telemedicine 

NICU without telemedicine (65%) was most prevalent before COVID-19, and telephone calls were the main (31%) communication method if implemented (Table 3). During COVID-19, the percentage of NICUs providing telemedicine combining telephone and online visits with neonates and communication with healthcare providers increased (from 2% to 19%).

### 3.3. NICU Design

In this study, the single-family room NICU design was not used, and we examined the association between NICU design (open bay or hybrid) with NICU visiting screening and available parental presence hours. Both NICU designs ensured significant increase in routine screening visits and decrease in the possible parental visiting time during COVID-19; however, less decrease was observed in these variables in hybrid NICUs than in open bay NICUs (Table 4).

### 3.4. Effect of Parental Restriction on Nursing Care and Parental Self-Refrain from Visiting

In 28 NICUs (54%), some nursing care could no longer be performed due to infection control compared to that before COVID-19. Table 5 shows the description of nursing care between pre-COVID-19 and during COVID-19. In 45 NICUs (86.5%), self-refrain from visiting occurred in 12 NICUs, with responders asserting that “*There may have been something I did not understand*.” In 26 NICUs (50%), a delay was observed in parents’ acquisition of childcare skills due to parental restriction, nursing care restriction, and self-restraint from visiting. In addition, in 19 NICUs (36.5%), there were responses of delay in the discharge of neonates caused by the aforementioned restrictions.

### 3.5. Association between Delayed Discharge during COVID-19 and NICU Design and Policies

We observed an association between delay discharge and parental presence policy. The proportion of NICUs that allowed the simultaneous entry of both parents into the NICU had fewer cases of delayed discharge than that allowed the entry of only one parent into the NICUs (Table 6). No association was observed between delay discharge and telemedicine and NICU design.

## 4. Discussion

The study results showed that all the NICUs that responded to the pandemic-imposed restrictions on the 24-h presence of parents and family members, and most patients in the NICUs were screened for mobility, poor physical condition, and body temperature (Table 3). The median parental presence allowed time was 1 h (range: 0–23 h). We found that 23 NICUs (44%) allowed only one parent at the bedside with limited time for the entire hospital stay and 11 NICUs (21%) allowed only mothers into the NICU. In 19 NICUs (36.5%), there were responses of delay in the discharge of neonates caused by the aforementioned restrictions. We also noted that there was a decrease in the implementation of skin-to-skin contact care during COVID-19 compared to that before COVID-19. Mothers frequently reported as not being supported to provide skin-to-skin care to their infant or encouraged to breastfeed as soon as possible after birth and inadequate information on expressing breastmilk or breastfeeding support during COVID-19 [5]. Similarly, our study found that the implementation rate of kangaroo care and milking decreased due to the COVID-19 pandemic (Table 5). It was speculated that the decrease in physical and emotional contact between parents and children and the lack of time to acquire childcare skills resulted in an extension of discharge from the NICUs.

In this study, the NICUs of hospitals allowing the simultaneous entry of both parents tended to have a longer median parental visit duration (2 h > 1 h; Figure 1). It was observed that NICU policies were implemented outside Japan during the pandemic [2,3,5], and the policy was associated in decreasing the incidence of delay in neonatal discharge from Japanese NICUs (Table 6). One study suggested that a policy of one parent entry restricted to limited duration was associated with a higher proportion of issues related to lack of bonding as well as the inability to participate in care, obtain updates, and bring supplies, followed by the entry of both parents with a restriction on the duration of presence [5]. Therefore, in the case of a parental presence time-limited policy, the presence of both parents at the same time might mitigate adverse effects. Moreover, this study showed that some NICUs limited entry to mothers only because based on Japan’s social background, mothers play a major role in childcare, and only 5.1% of fathers take childcare leave [10]. Many fathers work after their children are born, which increases their infection risk. 

In addition, NICUs with hybrid design allowed longer parental visit time than those with multi-bed rooms (Table 4). The Japanese level III NICU design criteria were the only established criteria for reimbursement. They require an area of 7 m^2^ per bed, an area smaller than the 11.2 m^2^ criterion of another NICU design [11]. In Japan, there are no standard designs for private NICU rooms. However, the outcomes of this study are consistent with the results of a previous study [3]. Thus, the merit of hybrid design NICU in maintaining physical distance between families was considered to mitigate strict parental presence restrictions.

Telemedicine was increasingly implemented to solve the problem of the loss of communication opportunities between parents and their neonates and healthcare providers (Table 3). However, the results of this study showed that telemedicine was not associated with a decrease in the incidence of neonatal discharge delay in Japanese NICUs (Table 6). In NICUs that responded, the new telemedicine facility was initiated on 4 August 2020, and the study only analyzed data on telephone or online telemedicine (Item No. 16 of Appendix A). Therefore, we believe that the extent of using telemedicine in Japanese NICUs was insufficient, and it is premature to conclude that telemedicine exclusively influenced the study results. Remote medicine allows parents to communicate with their children and express their feelings. This connection is through the parents’ smartphones, tablets, PCs, and NICU information devices through online video calls [4], virtual visits [6], mobile apps [12], and web camera systems [13,14,15,16,17,18]. In previous studies, parents watching live videos from a webcam felt separated from their children [16] and experienced the pain of helplessness in alleviating their children’s suffering [16,17]. However, many positive opinions from parents watching their children’s live videos were also reported. Owing to advanced medical technology, which enabled less stressful contact between parents and their children, parents felt close to their children [17] and could confirm the stability of their children’s condition [17,18]. Nevertheless, the confidentiality of patients’ information was a major concern for both parents and healthcare professionals because of the transmission of live videos [17,18]. The need to create communication materials for medical staff and parents through videos [15,17] increased the workload of nurses [15]. However, they thought that parents could see and understand their children’s responses immediately after birth by watching their neonates’ videos to promote bonding between parents and children [13,17]. Thus, remote medicine is considered a useful means of mitigating the adverse effects of the isolation of parents and children, despite parent restriction from NICU entry due to COVID-19.

One of the limitations of this study is the low response rate. The survey was conducted at 109 institutions (110 centers, of which one declined); however, only approximately half of them responded. The preferred response rate was ≥60% [19]. In addition, the result of this study was extrapolated from nurses’ perception. Because the outcomes of this study were at the parent-level, a survey conducted directly on parents to assess their satisfaction with telemedicine and its ability to help alleviate the negative impact of NICU in-person visiting restrictions should have been included. Finally, this study is a one-time cross-sectional study of hospitals and does not consider the medical condition or background of individual patients. Furthermore, the hospital’s pandemic policy may have influenced the extension of length of hospital stay. Therefore, future research should examine whether COVID-19 parental admission restrictions are associated with extended hospital stays for newborns, even when adjusted for patient attributes and institutional background. However, it is remarkable that the study clarified the parental visit restrictions in level III NICUs of approximately half of the Japanese population. This study suggests that the parents of NICU patients in Japan are relatively strictly restricted from entering the hospital after the COVID-19 outbreak. The University of Oxford [20] showed the assessment of the severity of countries’ COVID-19 policies based on nine perspectives on a 0–100 scale (100 being the strictest score). The highest score of Japan’s policies strictness from January to December 2020 was 40 (March to May 2020), which is moderate compared to that of other countries. However, the strict control of the visits of hospitalized patients’ families to the hospitals was in effect nationwide. Remarkably, restrictions on parental presence in the NICU were consistent with the hospitals’ policy. The results of this study raise policy and ethical questions about the separation of parents and their neonates in the NICU, which is expected to continue in Japan due to delays in COVID-19 vaccination, and provide a basis for studying effective measures to mitigate the effects of separation between parents and their babies.

One might argue that concerns remain regarding the generalizability of the observations of this study on NICUs in other locations worldwide. We acknowledge this weakness; however, to the best of our knowledge, findings in NICU studies are usually limited to the specific region (e.g., country) wherein the study was conducted [2,3,5]. This might be because the condition of the NICUs would clearly differ across regions owing to differences in political and economic aspects. The nature of the current study was not to compare the condition of NICUs across multiple countries to clarify any cross-culturally universal principles. Alternatively, it aimed to describe the parental visit policy and telemedicine use in Japanese neonatal intensive care units (NICUs) before and during the coronavirus disease (COVID-19) pandemic. Additionally, we investigated the effects of parental restriction on parental infants’ care, as well as the association between delay discharge and NICU design, telemedicine and parental visiting policy, which were reported to be a useful method for mitigating the negative impact on parents. We believe that the current findings not only provide evidence to the academic community, which helps to understand the complete picture of the NICU issues worldwide, but also influences the governments of the related regions to improve political practices over time.

## 5. Conclusions

Many NICUs began strict parental restrictions on entry into the NICU from April 2020 due to the COVID-19 pandemic. This restricted parents from entering the hospital and reduced their time spent in the NICU. The implementation rate of kangaroo care and milking was found to decrease due to the COVID-19 pandemic. Those restrictions might be associated with an extension of neonatal discharge in some NICUs. Further research is necessary to evaluate the clinical effects of these restrictions on the bonding between parents and children, the development of newborns, and the lengths of hospital stay. NICUs with hybrid design were able to lengthen parental presence and mitigate parental restrictions. In addition, in the case of a time-limiting parental presence policy, the presence of both parents at the same time might mitigate adverse effects. In Japan, the development and introduction of telemedicine utilizing web camera systems, virtual space, and mobile apps in the NICU are required due to currently inadequate the use of telemedicine. Regardless of COVID-19, it is necessary to consider the needs of parents who cannot enter the NICU when developing an appropriate telemedicine method. Further research is necessary to evaluate the feasibility and acceptability of telemedicine among both nurses in NICU and parents. 

## Figures and Tables

**Table 1:** Background of NICUs (*n* = 52).

Number of NICU hospital beds, median (range)	15	6–36
Acceptance of pregnant women and newborns newly infected with coronavirus		
Hospital-born only (*n*, %)	11	21.1
Nosocomial and out-of-hospital (*n*, %)	41	78.8
Only out-of-hospital born (*n*, %)	0	0

NICU, neonatal intensive care units; SD, standard deviation.

**Table 2:** Reasons for parental restriction in the NICUs.

	*n* (%)
Local policy restriction	21 (40)
Hospital policy restriction	45 (87)
Parental cohabitant had a risk of infection due to contact with an unspecified number of people on outings	16 (31)
Parent is infected/suspected of the novel coronavirus disease	14 (27)
Parental cohabitant with/suspected of novel coronavirus disease	12 (23)
Parental cohabitant stays in areas at high risk of infection	12 (23)
Parental cohabitant had a risk of infection from the collective life	10 (19)
Parental cohabitant with a high risk of becoming critically ill	4 (8)

Note: NICU, neonatal intensive care units.

**Table 3:** Comparison of NICU policies before and during COVID-19.

	Pre- COVID-19		During COVID-19		
	*n* = 52		*n* = 52		
	No.	(%)	No.	(%)	*p*
Confirmation of parent’s history of stay and transfer	2	4	42	81	0.021 *
Checking parents’ physical condition	35	67	51	98	<0001 *
Parental temperature measurement	7	13	50	96	0.096 *
Parents can enter the room for up to 24 h	22	42	0	0	0.001 *
Parental presence possible time, h, median (range)	12	(2, 24)	1	(0, 23)	<0001 ^§^
Family members other than parents can enter the room					<0001 ^†^
Grandparents only	26	50	2	4	
Siblings only	2	4	0	0	
Grandparents and siblings	15	29	0	0	
Not possible	9	17	50	96	
Telemedicine					0.033 ^†^
Telephone call visit	16	31	11	21	
Online visits	1	2	5	10	
Telephone + online	1	2	10	19	
Not performed	34	65	26	50	

Note: * McNemar’s test, ^§^ Wilcoxon test and ^†^ Cochran-Armitage test. COVID-19, coronavirus disease; NICU, neonatal intensive care unit.

**Table 4:** Changes in NICU entry screening and visiting time by NICU designs.

	Open Bay Design				Hybrid Design			
	*n* = 45				*n* = 7			
	Pre- COVID-19		During COVID-19		Pre-COVID-19		During COVID-19	
	*n*	%	*n*	%	*n*	%	*n*	%
Screening questions for travel history	2	4	37	82	0	0	5	71
Screening questions for illness	29	64	44	98	6	86	7	100
Screening temperature check	6	13	43	96	1	14	7	100
Parental always welcome (24 h)	19	42	0	0	3	21	0	0
Parental presence possible time, h, median	12	(2, 24)	1	(0, 18)	20	(2, 24)	4	(0.5, 23)

Note: COVID-19, coronavirus disease; NICU, neonatal intensive care unit.

**Table 5:** Comparison of nursing care between pre-COVID-19 and during COVID-19.

	Pre- COVID-19		During COVID-19	
	*n* = 52		*n* = 52	
	No.	(%)	No.	(%)
Kangaroo care	50	96	36	69
Parental hug	51	98	49	94
Parental touching	52	100	49	94
Breastfeeding	51	98	49	94
Parental bottle feeding	52	100	50	96
Milking in the units	51	98	46	89
Parental bathing	51	98	50	96
Parents changing diapers	52	100	51	98
Rooming in	33	63	30	58
Parent–nurse exchange diary	33	63	33	63
Recorded parent’s voice	3	6	3	6
Place a cloth with the scent of parents near neonates	21	40	21	40
Place parent and family photos near neonates	37	71	37	71
Providing parent videos of neonates	2	4	2	4
Providing parent photos of neonates	27	52	27	52
Siblings visit	25	48	11	21
Grandparents visit	42	81	21	40
Peer support between parents	9	17	6	12
Mental support from psychologists and social workers	45	87	45	87

Note: COVID-19, coronavirus disease.

**Table 6:** Association between delayed discharge during COVID-19 and NICU design and policies.

	Delay Discharge
	*n* (%)
	*p* *
NICU design	0.242
Open bay design, *n* = 45	18 (40)
Hybrid design, *n* = 7	1 (14)
Telemedicine	0.221
Telephone call visit, *n* = 11	3 (27)
Online visits, *n* = 5	4 (80)
Telephone + online, *n* = 10	4 (40)
Not performed, *n* = 26	8 (31)
Both parents entered the NICU simultaneously	0.047
Yes, *n* = 29	7 (24)
No, *n* = 23	12 (52)

Note: * Fisher’s exact test. NICU, neonatal intensive care unit; COVID-19, coronavirus disease.

## Appendix A. Web Survey of Parents’ Admission Restriction in NICUs

**<Consent to participate in research>** (one item)

1. Do you understand the content of the research description and agree to participate in this study? Yes/No

**<NICU demographics>** (six items)

2. Please select a location. Hokkaido/Tohoku/Kanto/Chubu/Kinki/Chugoku/Shikoku/Kyushu
3. Was your facility applicable to 13 prefectures that showed significantly increasing COVID-19 patients compared to other prefectures in April 2020? Yes/No
4. Which description accurately describes your unit about acceptance of patients with COVID-19? Inborn only/Inborn and transported admission/Out born only
5. Number of NICU beds
6. Number of GCU beds
7. Which design accurately describes your unit?
  Open Bay: 90% or more of beds in room.
  Hybrid: 11–89% of beds in single-patient rooms.
  Single-Family Room: 90% or more of beds in single-patient rooms.

**<Visiting policies and nursing care to bond parent and neonates before COVID-19>** (three items)

8. What was your Pre-COVID (December 2019) NICU visiting policy?
  (1) Screening question for travel history? Yes/No
  (2) Screening question for fever or illness? Yes/No
  (3) Screening for temperature? Yes/No
  (4) Parents always welcome (24 h)? Yes/No

If you answered “No,” how many hours per day were parents able to visit the NICU? hours
  (5) Was it possible for non-parental families to enter the NICU? Only grandparents/Only siblings/Both/No possible

9. From the following, select the care that was provided for bonding between parents and children. [Multiple answers allowed]

Kangaroo care/Parental hug/Parental touching/Breastfeeding/Parental bottle feeding/Milking in the units/Parental bathing/Parents changing diapers/Rooming in/Parent–nurse exchange diary/Recorded parent’s voice/Place a cloth with the scent of parents near neonates/Place parent and family photos near neonates/Providing a video of neonates/Providing photos of neonates/Siblings visit/Grandparents visit/Peer support between parents/Mental support from psychologists and social workers

10. What was your Pre-COVID telemedicine approach? [Multiple answers allowed]

Online visit/Telephone call visit/Not implement
**<Visiting policies and nursing care to bond parent and neonates after COVID-19 pandemic>** (6 items)

11. When did your NICU change its visiting policy? Date:
12. Which of the following reason(s) was/were applicable for parental restriction in your NICU? (Multiple answers allowed)

Policy of restricting visits to hospitals/ Parent is infected/suspected of the new coronavirus infection/ Parental cohabitant suspected of the new coronavirus infection/ Parental cohabitant had a risk of infection from work or school/ Parental cohabitant with a high risk of becoming critically ill/Parental cohabitant had a risk of infection due to contact with an unspecified number of people on outings/Declaration of emergency situations and requests from local governments to refrain from going out coronavirus/Parents living with new coronavirus infected/suspected patient

13. What is your current COVID NICU visiting policy? Same as 8. (1)–(5)
  (6) Can parents enter the NICU simultaneously? Yes/No
  (7) If you answered “No,” which policy accurately describes of your unit? Parents can enter the unit separately/Only those who decide which one can enter/Only mothers can enter/Only fathers can enter
14. From the following, select the care provided for forming a bond between the parents and the children. (Multiple answers allowed) Same as 9.
15. What is your current COVID telemedicine approach? Same as 10.
16. When did you start telemedicine during COVID-19? Date:

**<Self-restrain from visiting>** (one item)

17. Did parents refrain from visiting your unit due to COVID-19? Yes/No/I don’t know/There may have been something I did not understand

**<Impact on acquisition of childcare skills and length of hospital stay>** (four items)

18. Did parental admission restrictions, nursing care restrictions, and self-restraint visits affect parents’ acquisition of childcare skills? Yes/No/I don’t know
19. If you answered “Yes,” Please describe in detail why.
20. Did parental admission restrictions, nursing care restrictions, and self-restraint visits cause delay in the discharge of neonates? Yes/No
21. If you answered “Yes,” Please describe in detail why

**<Future infectious disease countermeasures and parent-neonates support>** (one item)

22. Until the COVID-19 countermeasures are established, please answer the perception of need for countermeasure to support parent and neonates in your unit with a seven-point Likert scale (1: Not needed at all; 7: Very needed)
  ● Admission screening by PCR test for parents
  ● Admission screening by antigen test for parents
  ● Admission screening by antibody test for parents
  ● Telemedicine using phone, smartphones, tablets, and PCs
  ● Early discharge support in collaboration with home-visit nursing
  ● Online visits within the family members
